# Choroidopathy and Retinal Detachment: A Rare Sighting in a Case of Postpartum Hemorrhage Presenting With Posterior Reversible Encephalopathy Syndrome

**DOI:** 10.7759/cureus.50731

**Published:** 2023-12-18

**Authors:** Nikhil Pantbalekundri, Swapneel Mathurkar, Neema Acharya, Sunil Kumar, Sourya Acharya

**Affiliations:** 1 Department of General Medicine, Jawaharlal Nehru Medical College, Datta Meghe Institute of Health Education and Research, Wardha, IND; 2 Department of Ophthalmology, Jawaharlal Nehru Medical College, Datta Meghe Institute of Health Education and Research, Wardha, IND; 3 Department of Obstetrics and Gynaecology, Jawaharlal Nehru Medical College, Datta Meghe Institute of Health Education and Research, Wardha, IND

**Keywords:** maintenance hemodialysis, hellp syndrome, posterior reversible encephalopathy syndrome, postpartum hemorrhage, choroidopathy

## Abstract

Posterior reversible encephalopathy syndrome (PRES) is a rare syndrome characterized by convulsions, headache, fatigue, impaired mental status, and decreased vision. It is mainly accompanied by hypertension. Although the pathophysiology of PRES is unknown, some theories revolve around cerebral autoregulation, the ability to maintain cerebral blood flow, or the brain's ability to maintain steady cerebral blood flow over a varying range of blood pressures by cerebral vaso-constriction or dilation. The presence of subcortical vasogenic edema in the posterior brain and hyperintensity lesions in the occipital and parietal lobes on magnetic resonance imaging (MRI) of the brain is diagnostic. We present the case of a woman who acquired PRES after a postpartum hemorrhage with no underlying disease, eventually leading to a choroidopathy and sudden onset diminution of vision, early diagnosis of which saved the patient from the catastrophic complication of permanent blindness.

## Introduction

Posterior reversible encephalopathy syndrome (PRES) is typically associated with altered mental status, seizures, and diffuse headaches [1]. The common risk factors include preeclampsia, hypertension in pregnancy, chronic liver or renal disease, exposure to hazardous drugs, biological immune suppressants, and autoimmune disorders [2]. Rarely, spinal cord injuries, hemoglobinopathies, and snake bites have been reported with PRES. Poorly managed hypertension is the most common of these etiologies or triggers. PRES occurring in patients without any history of systemic hypertension are rarely reported [3,4]. PRES can present acutely with varying symptoms ranging from hours to days. The underlying pathology of the disease is unknown however based on current knowledge, it is hypothesized that it is a disorder of cerebral autoregulation. The mainstay treatment of PRES is supportive, correction of underlying causes, and management of complications. Most patients recover from PRES; however, it is not always reversible and is associated with significant morbidity and mortality. Choroidal hypo-perfusion is a critical ocular consequence of postpartum hemorrhage (PPH) [5]. Here, we present a case of a young full-term primigravida who underwent an emergency cesarean section and developed PRES syndrome. It was followed by a sudden diminution of vision, which on fundoscopic examination revealed choroidopathy and serous retinal detachment as a consequence of postpartum hemorrhage.

## Case presentation

A 24-year-old female, primigravida with no previous history of hypertension or diabetes underwent an emergency cesarean section in the 39th week for non-progression of labor and delivered a male baby of 3.7 kg. She had undergone thorough antenatal check-ups throughout her pregnancy, which was uneventful. Postoperatively, the patient developed postpartum hemorrhage. Uterine balloon tamponade was placed. Intravenous pitocin, intramuscular methargin, and tablet misoprostol 800 mg rectally were administered, and balloon tamponade was removed after 3 hours. The tone was achieved and the bleeding was stopped. Three units of packed red cells were transfused over the span of 2 days and the patient was referred to our hospital because of oliguria for the last 24 hours (100 ml/24 hrs). On examination, blood pressure was 150/90 mmHg, heart rate was 90/min, and the patient had pallor and pedal edema. On auscultation, fine crepitations were heard in bilateral infra-scapular and infra-axillary regions. All the routine blood investigations are highlighted here (Table 1).

**Table 1:** Investigation on admission.

Investigations	Value	Normal
Kidney function test		
Serum urea	120 mg/dl	6-24 mg/dl
Serum creatinine	8.2 mg/dl	0.7-1.3 mg/dl
Serum sodium	136 mmol/l	135-145 mmol/l
Serum potassium	5.9 mmol/l	3.5-5.1 mmol/l
Liver function test		
Serum alkaline phosphatse	151 IU/L	90-300 IU/L
Serum glutamate pyruvate transaminase	136 IU/L	0-45 IU/L
Serum glutamate oxaloacetic transaminase	153 IU/L	0-50 IU/L
Albumin	2.2 gm/dl	3.5-5 gm/dl
Total protein	4.3 gm/dl	6-8.5 gm/dl
Total bilirubin	1.1 mg/dl	0-1 mg/dl
Conjugated bilirubin	0.4 mg/dl	0-0.35 mg/dl
Unconjugated bilirubin	0.7 mg/dl	0-0.65 mg/dl
Complete blood count		
Hemoglobin	10 gm/dl	13-17 gm/dl
White blood cells	28300/cmm	4500-10500/cmm
Platelets	100000/cmm	150000-400000/cmm
International normalized ratio	1.4	0.8-1.2
Serum calcium	8.6 mg/dl	9-11 mg/dl
Serum magnesium	1.4 mg/dl	1.6-2.5 mg/dl
Serum uric acid	7 mg/dl	3.5-7.2 mg/dl
Serum lactate dehydrogenase	5136 units/l	150-300 units/l

Arterial blood gas was suggestive of metabolic acidosis. Ultrasonography (USG) of the abdomen and pelvis showed the average size of kidneys and raised echotexture. Based on the peripheral smear report suggestive of few fragmented RBCs, elevated serum unconjugated bilirubin and elevated serum lactate dehydrogenase (LDH) levels, and thrombocytopenia, a provisional diagnosis of HELLP (hemolysis, elevated liver enzymes, low platelet count) syndrome with acute kidney injury was made. The patient was started on intravenous piperacillin-tazobactam 2.25 gm thrice a day, intravenous levofloxacin 500 mg once every alternate day, intravenous amoxicillin-clavulanate 1.2 gm twice a day, intravenous frusemide 40 mg twice a day along with supportive medication. Tab nicardipine 20 mg thrice a day was added because of persistent hypertension.

**Figure 1:**
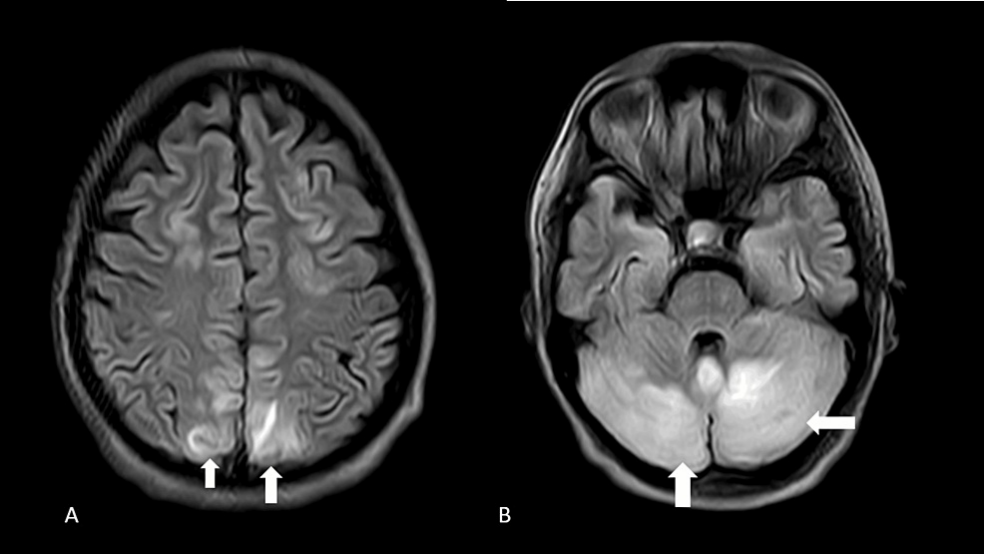
MRI brain; T2 FLAIR sequence showing cortical white matter signal hyperintensity foci (white arrows) in bilateral occipital, parietal regions (A) and bilateral cerebellar hemispheres (B). FLAIR: Fluid attenuated inversion recovery

On the second day of admission, the patient had two episodes of convulsion lasting for up to 2-3 minutes, followed by unconsciousness. The patient had normal blood sugar levels and the electrolyte panel was within the normal limits. The patient complained of sudden onset diminution of vision, continuous headache, pedal edema, and significant facial puffiness. A brain CT suggested subcortical hypo-densities in the bilateral occipito-parietal region and watershed frontal and temporal lobe areas with cerebral and cerebellar edema. With suspicion of PRES syndrome, an MRI brain was ordered. T2 FLAIR (fluid-attenuated inversion recovery) sequences of MRI brain showed signal hyperintensity in white matter regions of bilateral occipital and parietal regions along with cerebellar hemispheres (Figure 1).

**Figure 2:**
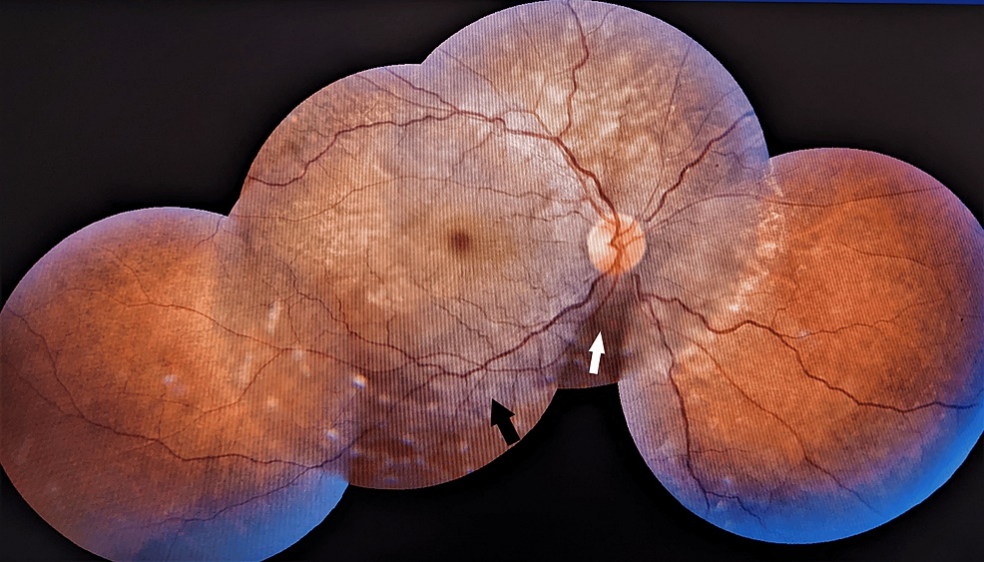
Right retina showing inferior serous retinal detachment involving upto macula (white arrow) with choroidal nodules (black arrow).

**Figure 3:**
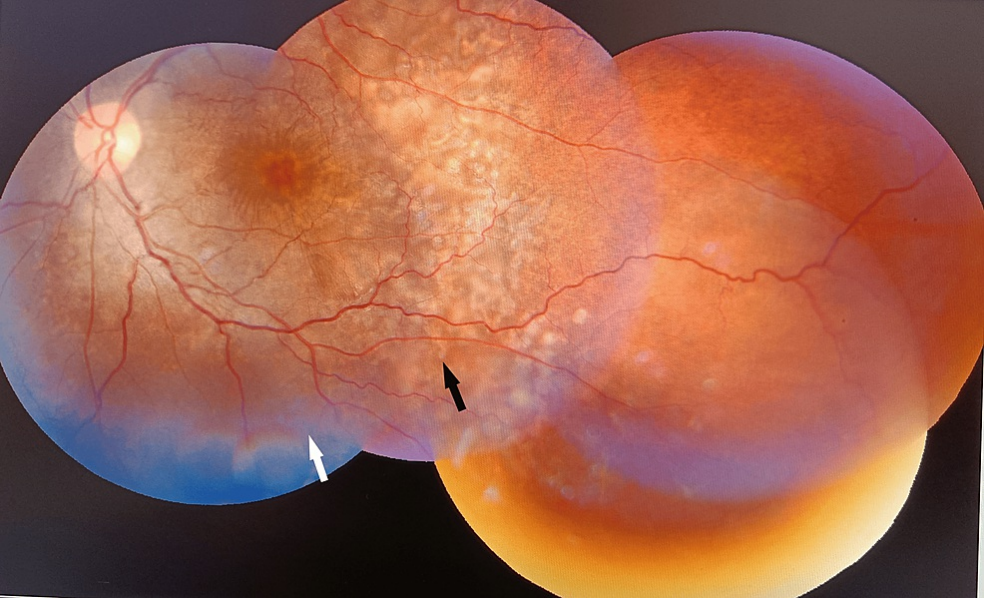
Left retina showing inferior serous retinal detachment (white arrow) and choroidal nodules (black arrow).

On ophthalmic examination both eyes showed disc hyperemia, margins were well-defined and disc cups were congested. Multiple whitish choroidal lesions over the posterior pole suggestive of choroiditis were evident. Tortuous retinal vessels and serous retinal detachment at the macula were seen (Figure 2, Figure 3).

**Figure 4:**
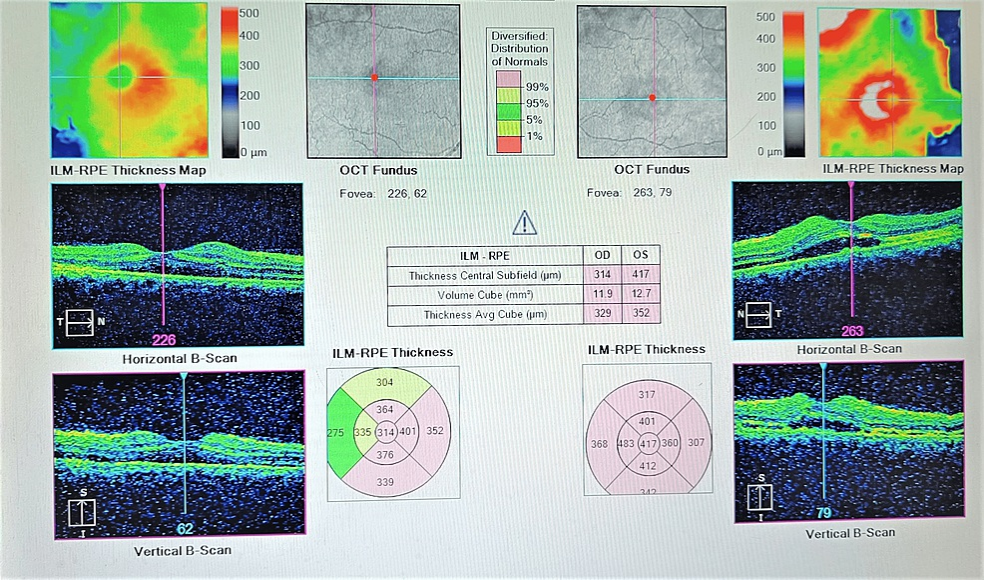
Ocular coherence tomography suggestive of bilateral serous retinal detachment.

Ocular coherence tomography (OCT) of both eyes showed sub-retinal fluid between the retinal pigment epithelial layer and neurosensory retinal layer at the macular region which confirmed serous retinal detachment (Figure 4).

With clinical correlation, a diagnosis of postpartum posterior reversible encephalopathy syndrome with choroidopathy in a patient with acute renal failure and newly diagnosed hypertension was made. Rapidly rising serum creatinine and blood urea levels were noted; co-relating with oliguria; along with resistant hyperkalemia on serial monitoring of electrolytes. With persistent metabolic acidosis, and nonresponding to intravenous correction, the patient was started on conventional intermittent hemodialysis. Intravenous meropenam 300 mg twice a day, intravenous clindamycin 600 mg twice a day, intravenous moxifloxacin 5 mg/ml, and dexamethasone 1 mg/ml eye drops were added. The patient's symptoms subsided, and clinical improvement was noted with consecutive hemodialysis and as blood pressure was controlled. Routine ophthalmic examination showed residual peripheral choroidal nodules with no visual impairment. Kidney function tests revealed improvement in serum creatinine and urea levels, and the patient was continued on conservative management.

## Discussion

Headaches, seizures, altered mentation, neurological deficit with the presence of risk factors like systemic hypertension, hypertension in pregnancy, autoimmune disorders, chronic liver or renal disease, etc., are suggestive of PRES. PRES can appear with symptoms anywhere between hours and days [6]. This clinical and radiographic syndrome is named after radiographic findings of white matter edema, typically found symmetrically in the posterior cerebrum. The symptoms of PRES are reversible; hence, early diagnosis and prompt management are vital to improve the patient's prognosis [7,8].

The pathophysiology of PRES is unknown; however, the widely recognized theory is of cerebral autoregulation, which is the ability to maintain cerebral blood flow over a varying range of blood pressures. When blood pressure is high, typically above 160 mmHg, the vasoconstriction required to maintain constant cerebral blood flow is at its peak, and blood flow begins to rise as blood pressure rises. Rising blood pressure can cause a blood-brain barrier breakdown, allowing intravascular fluid to ooze into the surrounding brain tissue and cause cerebral edema [9,10]. Blood transfusion may result in a rapid increase in blood volume, resulting in increased cerebral flow [11]. Vasogenic edema in PRES may result from acute cerebral hyper-perfusion that exceeds the limits of auto-regulation. Severe anemia, therefore, is a pre-disposing factor due to insufficient oxygen supply to the brain, which may result in endothelial cell dysfunction, causing damage to the blood-brain barrier in capillary circulation, thus causing cerebral edema. Hemodialysis lowers plasma osmotic pressure by allowing harmful elements from the blood, such as creatinine, urea, and nitrogen, to diffuse into the hemodialysis solution. Simultaneously, the extracellular fluid gradually decreases due to ultrafiltration, increasing the extracellular fluid's osmotic pressure. The extracellular osmotic pressure is then buffered by altering intracellular fluid outflow, which leaves tissues with a relative water deficit. Reduced choroidal and retinal edema may result from hemodialysis's effects on the thickness of the eye coats and the vitreous axial length, which includes the thickness of the ciliary body, choroid, and retina. A brief drop in ocular perfusion pressure occurs during hemodialysis. In this case, hemodialysis was primarily used to treat acute renal failure resistant to medical strategies [12]. There has been less research on the effect of hemodialysis on choroidal perfusion dysregulation in patients with acute kidney injury or chronic kidney disease. The impact of blood pressure variations during hemodialysis on choroidal perfusion requires more investigation. In our case, the ocular symptoms probably improved due to prompt management of PRES and PPH.

The effect of hypovolemic shock during PPH is related to the mechanism of PRES with PPH without a history of hypertension [13]. On interpretation, ischemic edema caused by vasoconstriction and hypo-perfusion is one hypothesis for the onset of PRES after PPH [14,15]. The hypovolemia caused by PPH with diversion of blood flow to critical organs may cause ischemic damage to ocular tissue and visceral organs. There is little reported literature on PPH-associated visual loss (PPHAVL). Choroidal hypo-perfusion is an unusual but significant ocular complication of PRES associated with PPH [16,17]. In our case, it was diagnosed early, and dreaded complications of retinal detachment and permanent blindness were prevented with early intervention.

## Conclusions

Posterior reversible encephalopathy syndrome (PRES) is a rare clinical-radiological syndrome marked by convulsions, headache, altered mental status, and decreased eyesight, and is usually accompanied by hypertension. PRES should always be considered in women presenting with acute hypertension, postpartum seizures, or other neurological symptoms. Choroidopathy and visual disturbances, although rare in PRES with postpartum hemorrhage should always be looked out for early diagnosis and prevention of permanent visual loss.

## References

[1] Triplett JD, Kutlubaev MA, Kermode AG, Hardy T (2022). Posterior reversible encephalopathy syndrome (PRES): diagnosis and management. Pract Neurol.

[2] Fugate JE, Rabinstein AA (2015). Posterior reversible encephalopathy syndrome: clinical and radiological manifestations, pathophysiology, and outstanding questions. Lancet Neurol.

[3] Shi F, Shen L, Shi Y (2017). Posterior reversible encephalopathy syndrome after postpartum hemorrhage and uterine artery embolization: A case report. Medicine (Baltimore).

[4] Marcoccia E, Piccioni MG, Schiavi MC (2019). Postpartum posterior reversible encephalopathy syndrome (PRES): three case reports and literature review. Case Rep Obstet Gynecol.

[5] Powell S, Garrahy D, Stephenson KA, Burke T (2022). Postpartum haemorrhage associated choroidopathy. BMJ Case Rep.

[6] Sudulagunta SR, Sodalagunta MB, Kumbhat M, Settikere Nataraju A (2017). Posterior reversible encephalopathy syndrome(PRES). Oxf Med Case Reports.

[7] Gao B, Yu BX, Li RS, Zhang G, Xie HZ, Liu FL, Lv C (2015). Cytotoxic edema in posterior reversible encephalopathy syndrome: correlation of MRI features with serum albumin levels. AJNR Am J Neuroradiol.

[8] Edlow JA, Caplan LR, O'Brien K, Tibbles CD (2013). Diagnosis of acute neurological emergencies in pregnant and post-partum women. Lancet Neurol.

[9] Babahabib MA, Abdillahi I, Kassidi F, Kouach J, Moussaoui D, Dehayni M (2015). Posterior reversible encephalopathy syndrome in patient of severe preeclampsia with Hellp syndrome immediate postpartum. Pan Afr Med J.

[10] Somani A, Gaidhane SA, Gaidhane PA, Khatib N, Acharya S (2021). Posterior reversible encephalopathy syndrome (PRES) in haemolytic anaemia - a case report. J Evolution Med Dent Sci.

[11] Dube M, Rathore R (2020). Blood-transfusion-related posterior reversible encephalopathy syndrome - a description of a new case and review of the literature. Brain Circ.

[12] Wang L, Yin G, Yu Z, Chen N, Wang D (2018). Effect of hemodialysis on eye coats, axial length, and ocular perfusion pressure in patients with chronic renal failure. J Ophthalmol.

[13] Ahuja A, Saboo K, Kumar S, Acharya S, Agrawal S (2023). Amaurosis fugax in posterior reversible encephalopathy syndrome: a vexed hurdle in a postpartum primigravida patient. Cureus.

[14] Reddy V, Saboo K, Kumar S, Acharya S, Patel DJ (2023). Beyond the shadows: unravelling the menace of methanol-induced posterior reversible encephalopathy syndrome. Cureus.

[15] Khurana K, Acharya S, Shukla S, Kumar S, Mishra P (2023). Chronic glomerulonephritis and malignant hypertension with PRES (posterior reversible encephalopathy syndrome) presenting as status epilepticus: a case report. Cureus.

[16] Karaguzel H, Guven S, Karalezli A, Erdol H (2009). Bilateral serous retinal detachment in a woman with HELLP syndrome HELLP syndrome and retinal detachment. J Obstet Gynaecol.

[17] Tranos PG, Wickremasinghe SS, Hundal KS, Foster PJ, Jagger J (2002). Bilateral serous retinal detachment as a complication of HELLP syndrome. Eye (Lond).

